# Notes from the Field: Phenibut Exposures Reported to Poison Centers — United States, 2009–2019

**DOI:** 10.15585/mmwr.mm6935a5

**Published:** 2020-09-04

**Authors:** Janessa M. Graves, Julia Dilley, Sanjay Kubsad, Erica Liebelt

**Affiliations:** ^1^Washington State University, College of Nursing, Spokane, Washington; ^2^Oregon Health Authority Public Health Division & Multnomah County Health Department, Portland, Oregon; ^3^University of Washington, School of Medicine, Seattle, Washington; ^4^Washington State Poison Center, Seattle, Washington.

Phenibut (*β*-phenyl-*γ*-aminobutyric acid) is an unregulated* drug developed in Russia in the 1960s for use as an antianxiety medication with cognitive enhancement properties (*1*). Online retailers recently have contributed to a growing U.S. market for phenibut, which is advertised for anxiety, relaxation, and sleep (*1*,*2*). Phenibut use and misuse can result in sedation, respiratory depression, and reduced levels of consciousness, as well as withdrawal symptoms including anxiety, agitation, and acute psychosis (*3*). Regional poison center data suggest that phenibut exposures have increased in recent years (*3*). To characterize the frequency of phenibut-related exposures in the United States, data on human exposure calls to U.S. poison centers during January 2009–December 2019 were extracted from the national database maintained by the American Association of Poison Control Centers.^†^

Phenibut exposures were identified as poison center calls involving human exposure to phenibut; searches included synonyms (i.e., phenygam or 4-amino-3-phenylbutyric acid)^§^ (*4*). Exposures do not necessarily represent a poisoning or overdose. All exposure calls involving single or multiple substances were included^¶^; calls requesting information on phenibut were not included. The analysis summarized the demographic characteristics, caller location (e.g., health care facility or residence), exposure routes, clinical health effects, and outcomes.

For each poison center call, a case record for a single exposure event (case) is generated, delineating the patient’s history, physical examination, clinical assessment, and recommendations provided. Health care providers (e.g., nurses, pharmacists, and physicians) provide ongoing case management through follow-up calls until the acute toxicologic condition has resolved; therefore, each case might involve more than one call. Multiple data elements are recorded (e.g., reason for poisoning, patient age, substances, clinical effects, therapies, and medical outcomes), as determined by the providers managing the exposures at each poison center. Health care providers managing cases identify the exposure agents by manufacturer name or synonym. Providers use standard National Poison Data System definitions to enable consistent reporting among poison centers and across years of data.

During 2009–2019, U.S. poison centers reported calls for 1,320 phenibut exposures from all 50 U.S. states and the District of Columbia. For most (1,122; 85.0%) cases, calls originated from health care facilities. Most exposures (58.4%) occurred among adults aged 18–34 years (mean = 31.7 years, standard deviation = 13.1 years, interquartile range = 22–38 years). The majority of reported exposures were in men (75.5%).

The number of cases increased sharply over the study period, particularly since 2015, when regional poison centers became able to use “phenibut” as a relevant term to capture exposures (Figure). Phenibut exposures with known formulations most often involved solids (e.g., tablets) (65.1%) or powder (24.8%). Reported exposures were predominantly ingestions (93.2%), although 2.8% involved inhalation, and 4.0% involved other routes of exposure, including dermal. Unintentional exposures were more common among persons aged <18 years (21.9%). A significantly higher percentage of exposures among children aged <10 years (93.3%) was unintentional, compared with 6.3% of those among adults (p<0.001). Coingested substances (i.e., exposure to more than one drug or agent) were reported in 29.6% of cases in persons aged <18 years and in 40.2% of all adult cases (p = 0.04).

**FIGURE:**
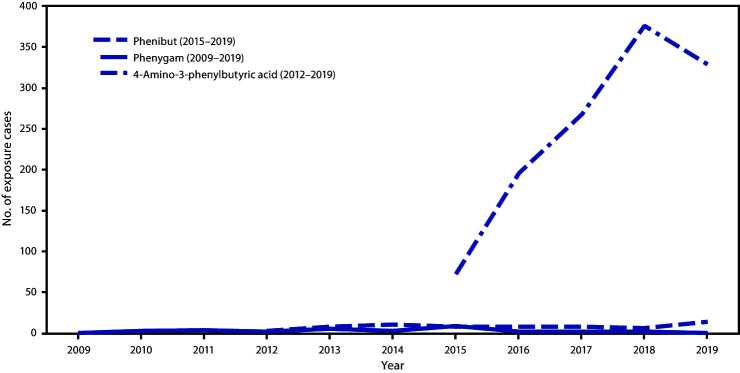
Number of human exposure cases related to phenibut use reported to poison centers, by year — National Poison Data System, United States, January 2009–December 2019

Commonly reported adverse health effects included drowsiness or lethargy (29.0%), agitation (30.4%), tachycardia (21.9%), and confusion (21.3%). Coma was reported in 80 (6.2%) cases, including one involving an adolescent. In one half (49.6%) of cases, the exposure resulted in moderate effects (i.e., no long-term impairment). Major effects (i.e., life-threatening or resulting in significant disability or disfigurement) occurred in one in eight (12.6%) reported exposures, and three deaths were reported. Among exposures in which phenibut was the only drug or agent involved, 10.2% were associated with major effects, including one death.

The reason for the increase in phenibut-related exposures during 2009–2019 is not known; growing popularity and availability of the product through online retailers might be contributing factors. The increase in phenibut exposures underscores the need for heightened awareness of phenibut as an emerging substance of use and misuse in the United States. Adverse health effects reported to poison centers, such as drowsiness or lethargy, agitation, and confusion, are consistent with those described in previous reports (*3*). Exposures were associated with long-term health effects, including death. Easy online access to phenibut (*2*) and the potential for dependence (*5*) are additional reasons for concern. Phenibut is uncontrolled and legal to possess in the United States. Educational efforts to increase awareness among the public and clinicians regarding the emerging popularity and dangers of phenibut might help prevent adverse health effects and outcomes, including death.

## Footnotes

§ Phenibut has been reported as “phenygam” in the National Poison Data System database since before the study timeline; “4-amino-3-phenylbutyric acid” and “phenibut” were added in 2012 and 2015, respectively.

¶ Phenibut exposures are not confirmed by laboratory testing; no commercially available test is available and phenibut is not detected on routine urine drug screens.
